# Liquid-Liquid Phase Transition and Glass Transition in a Monoatomic Model System

**DOI:** 10.3390/ijms11125184

**Published:** 2010-12-16

**Authors:** Limei Xu, Sergey V. Buldyrev, Nicolas Giovambattista, H. Eugene Stanley

**Affiliations:** 1 WPI Advanced Institute for Materials Research, Tohoku University, 2-1-1 Katahira, Aoba-ku, Sendai 980-8577, Japan; 2 Department of Physics, Yeshiva University, 500 West 185th Street, New York, NY 10033, USA; E-Mail: buldyrev@yu.edu; 3 Department of Physics, Brooklyn College of the City University of New York, Brooklyn, NY 11210, USA; E-Mail: ngiovambattista@brooklyn.cuny.edu; 4 Center for Polymer Studies and Department of Physics, Boston University, Boston, MA 02215, USA; E-Mail: hes@bu.edu

**Keywords:** glass transition, polyamorphism, liquid-liquid phase transition

## Abstract

We review our recent study on the polyamorphism of the liquid and glass states in a monatomic system, a two-scale spherical-symmetric Jagla model with both attractive and repulsive interactions. This potential with a parametrization for which crystallization can be avoided and both the glass transition and the liquid-liquid phase transition are clearly separated, displays water-like anomalies as well as polyamorphism in both liquid and glassy states, providing a unique opportunity to study the interplay between the liquid-liquid phase transition and the glass transition. Our study on a simple model may be useful in understanding recent studies of polyamorphism in metallic glasses.

## 1. Introduction

The phenomenon of polyamorphism of a single-component system has been receiving a considerable attention [1–3] since the observation of two or more distinct glasses in water [4–12]. A number of new substances of such laboratory transformations have been reported [13,14] including elemental [15–17], molecular [18], ionic [19], and covalent [20] systems. Recently, a metallic glass case, based on cerium [19], has been added to the list. Most of the experimental studies on polyamorphism involve transitions from an initial liquid state to either a second metastable liquid or to a glass. Polyamorphism in equilibrium, *i.e.*, a liquid-liquid phase transition (LLPT) [21], has been studied for bulk phosphorus [15,16], and interpreted to underlie experimental observation in bulk water [7], quasi-two-dimensional confined water, [22,23] and quasi-one-dimensional confined water [24–28], as well as in the thin layer of water surrounding biomolecules such as lysozyme, DNA, and RNA [25,29]. There is evidence from several sources [30–36] that the two liquid phases involved in a LLPT have rather different properties. Thus, the existence of a single-component system with two distinct glassforming liquid phases provides a rare opportunity to study the fundamental aspects of glass formation. However, it can be challenging to establish such properties unambiguously because of the propensity of crystallization of the low-entropy liquid. It is therefore of interest to find a model system in which liquid phases can be studied under stable as well as metastable conditions, and glass transition (GT) can be observed independently.

**Figure 1.**
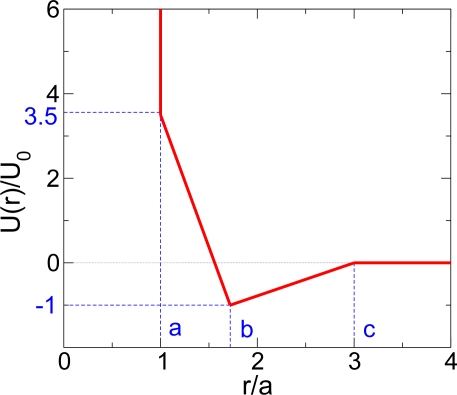
The spherically-symmetric “two-scale” Jagla ramp potential. The two length scales of the Jagla potential are the hard core diameter *r* = *a* and the soft core diameter *r* = *b*. Here we treat the case with *U_R_* = 3.56*U*_0_, *b* = 1.72*a*, and a long range cutoff *c* = 3*a*.

One such a model is the spherically-symmetric two-scale Jagla potential with both replusive and attractive interactions [30,37–42]. In this review, we will show that the two-scale Jagla potential (Figure 1), with a choice of parameters that crystallization can be avoided, exhibits polyamorphism not only in the equilibrium liquid phase at high temperature [30], but also in the glass states at low temperature [43]. It has been suggested that such spherically-symmetric potentials provide a generic mechanism for LLPT [44–48], and have interested experimentalists to seek examples among the liquid metals [19]. For example, Stell and coworkers had identified Cesium and Cerium as candidate systems [49–52], and indeed irreversible density changes under high pressure in glassy metals containing a large mole fraction of Ce have subsequently been reported [19]. Thus, the Jagla potential provides an excellent example of a very simply constituted system that is a good glassformer and it is suitable for pursuing the study of glassforming ability, which is obviously a key issue in the science of bulk metallic glasses. This resulted in a unique system that has allowed us to study the relation between the GT and the LLPT, which might be useful for the prediction of the relations between high density and low density metallic liquid phases that might be found in future studies of cerium-rich bulk metallic glassformers.

In this article, we will review our recent studies on a simple Jagla model of monoatomic system [34,35,37–39,43] as shown in Figure 1. It shows polyamorphism both in liquid and glasses and can be of interest to general understanding of polyamorphism in liquids (such as water, Yttrium Oxide-Aluminum [53]), and polyamorphism in metallic glasses (such as Ce-based alloys [19]).

## 2. Polyamorphism in the Liquid States

We firstly review the equilibrium properties of the liquid phases of the Jagla model, which is relevant to the study of liquid water. As we know, water is the most important solvent for biological function [54–56], yet water possesses many properties that are not well understood. One current hypothesis concerns the possibility that water’s anomalies are related to the existence of a line of a first order LLPT terminating at a liquid-liquid critical point (LLCP) [4,7,21,44,57], which is located in the deep supercooled region of the phase diagram below the homogeneous nucleation line, sometimes called the “no-man’s land” because it is difficult to make direct measurements on the *bulk* liquid phase [7]. For instance, the thermodynamic properties (e.g., specific heat) [58,59] of the bulk liquid water seems to diverge to a temperature (*T* ≈ 228 K) within the no man’s land region [6]. By confining water in hydrophilic nano-geometries, the liquid water can be stabilized down to much lower temperatures, which allows the detection of the thermodynamic property (e.g., specific heat), instead of divergence, exhibiting a maximum [60]. More recent experiment on nano-confined water by quasi-electric neutron scattering (QENS) and nuclear magnetic resonance (NMR) [24,61,62] showed that water appears to have dynamic crossover, between non-Arrhenius (“fragile”) behavior at high T to Arrhenius (“strong”) behavior at low T [62–66]. This indicates that the LLPT may have a strong effect on the dynamic properties of supercooled water, including the glass transition [67–71]. We note that, in this review, we only focused on one of four possible scenarios to explain the anomalous properties of water [72]. In particular, the *singularity-free* scenario [73–75] hypothesizes that the low *T* anticorrelation between volume and entropy is sufficient to cause the response functions to increase upon cooling and display maxima at non-zero *T*, without reference to any sigular behavior. In the first part, we focus on the relation between a liquid-liquid phase transition and the thermodynamic and dynamic properties [25,30,62,65,76–78].

### 2.1. Liquid-Liquid Phase Transition

**Figure 2.**
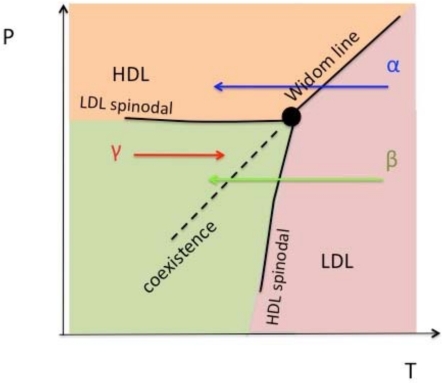
Sketch of the Jagla potential *P* – *T* phase diagram [30]. The low-density liquid (LDL) and high density liquid (HDL) phases are separated by a first order transition line (dashed line), terminating at a critical point at *P_c_* = 0.243 and *T_c_* = 0.373. The Widom line *T*_W_ indicates the locus of maxima in the correlation length that occurs in supercritical region (*T* > *T_c_* and *P* > *P_c_*). Studies in this work are along three different kinds of paths: (i) for *P* > *P_c_*, path *α* in the one phase region, (ii) for *P* < *P_c_*, path *β* in LDL phase, and (iii) for *P* < *P_c_*, path *γ* in HDL phase.

With the proper choice of parameters, as shown in Figure 1, the system shows a LLPT with a critical point located at *T_c_* = 0.375, *P_c_* = 0.243 and *ρ_c_* = 0.37 above the melting line [30,34,35]. The coexistence line, determined by Maxwell rule via integrating of the isotherms in the *P* – *V* phase diagram, shows a positively sloped liquid-liquid coexistence line [30]. A sketch of the phase diagram is shown in Figure 2. According to the Clapeyron equation,

(1) $$\frac{\text{dP}}{\text{dT}}=\frac{\Delta S}{\Delta V}$$

the entropy in the low temperature phase, high-density liquid (HDL), is lower than the high temperature phase, low-density liquid (LDL), due to a positively sloped coexistence line. Hence, the HDL phase is more ordered than the LDL phase, which is the opposite of the liquid-liquid transition found in simulation for water [65] and silicon [31].

The limit of stability of the less-ordered LDL phase is determined by the high pressure LDL spinodal *P*_LDL_(*T*), which, for our model, is unlikely to be crossed by cooling the system at constant pressure since *P*_LDL_(*T*) ≈ *P_c_* for all *T* except in the immediate vicinity of the LLCP. On the other hand, the limit of stability of the more ordered HDL phase is determined by the low pressure HDL spinodal *T*_HDL_(*P*), which can be crossed by heating the HDL phase at constant pressure. That is why the dynamic behavior of the more ordered HDL phase can be studied only when *T* < *T*_HDL_(*P*) for *P* < *P_c_* [34,35].

### 2.2. Liquid-Liquid Transition and the Widom Line

**Figure 3.**
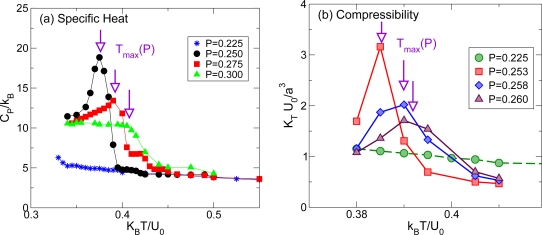
(Color online) Response functions for the Jagla ramp model as function of temperature for different values of *P* > *P_c_* (Figure 2, path *α*) and *P* < *P_c_* (Figure 2, path *β*). (a) Constant pressure specific heat *C_P_* and (b) isothermal compressibility *K_T_*. Both *C_P_* and *KT* have maxima, as is known to occur experimentally for the liquid-gas critical point [79] and for the LLCP [60]. For large *P* the peaks become less pronounced and shift to higher temperature as the Widom line has positive slope. Adapted from Reference [34].

If the system is cooled isobarically along a path above the liquid-liquid critical pressure *P_c_* (Figure 2, path *α*), the state functions continuously change from the values characteristic of a high temperature phase (LDL) to those characteristic of a low temperature phase (HDL). The thermodynamic response functions which are the derivatives of the state functions with respect to temperature, e.g., isobaric heat capacity (Figure 3(a)) and isothermal compressibility (Figure 3(b)), have maxima at temperatures denoted by *T*_max_(*P*). Remarkably these maxima are still prominent far above the critical pressure, as in the case of the liquid-gas critical phenomenon of water in Refs. [79–82], and the values of the response functions at *T*_max_(*P*) (e.g., $C_{P}^{\text{max}}$ and $K_{T}^{\text{max}}$) diverge as the critical point is approached. The lines of the maxima *T_max_*(*P*) for different response functions are different but asymptotically approach one another as the critical point is approached, since all response functions become expressible in terms of the correlation length. This asymptotic line is sometimes called the Widom line, and is often regarded as an extension of the coexistence line into the “one-phase regime”.

If the system is cooled at constant pressure below *P_c_* within the two phases region (Figure 2, path *β*), the coexistence line can be difficult to detect in a pure system due to metastability, and changes will occur only when the spinodal is approached where the initial phase is no longer stable. The response functions—*C_P_* (Figure 3(a)) and *K_T_* (Figure 3(b))—increase continuously along path *β* (Figure 3) before the system reaches the stability limit near the LDL spinodal [57].

### 2.3. Structural Changes and Liquid-Liquid Phase Transition

The structural properties [34] can be characterized by the translation order parameter and orientation order parameter. The translational order parameter *t* [88 – 83] is defined as $t\equiv \int_{0}^{r_{c}}\left|g(r)-1\right|dr$, where *r* is the radial distance, *g*(*r*) is the pair correlation function, and *r_c_* = *L*/2 is the cutoff distance, where *L* is dimension of the system. A change in the translational order parameter indicates a change in the structure of the system. For uncorrelated systems, the interaction in the system is short-ranged with *g*(*r*) = 1, leading to *t* = 0; for long-range correlated systems, the modulations in *g*(*r*) persist over large distances, causing *t* to grow.

The orientational order parameter characterizes the average local order of the system [83]. For each particle, there are 12 bonds connecting the centeral particle with each of its 12 nearest neighbours and each bond is characterized by two angles (θ, φ). For the *ith* particle, the orientational order parameter, *Q_l,i_*, is defined as

(2) $$Q_{\ell ,i}\equiv {\left[\frac{4\pi }{2\ell +1}\sum_{m=-\ell }^{m=\ell }{\left|{\bar{Y}}_{\ell ,m}\right|}^{2}\right]}^{1/2}$$

where *Ȳ_ℓ,m_*(*θ, φ*) denotes the average of the spherical harmonic function *Y_ℓ,m_*(*θ, φ*) over the 12 bonds associated with particle *i*. The orientational order parameter for the entire system is calculated as *Q_ℓ_* =< *Q_ℓ,i_* >, where < ... > denotes the average over all particles in the system. For ℓ = 6, *Q_ℓ_* has large value for most crystals, such as fcc, hcp, and bcc. In general, the value of *Q*_6_ increases as the local order of a system increases, e.g, *Q*_6_=0.574 for the fcc lattice and *Q*_6_ = 0.289 for uncorrelated systems.

**Figure 4.**
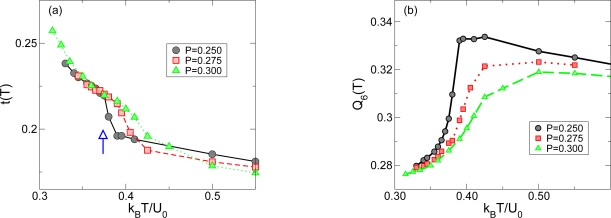
Structural changes upon crossing the Widom line region. The translational order parameter *t* (a) and the orientational order parameter *Q*_6_ (b). There is a sharp change in *t* and *Q*_6_ occurs as the system crosses the Widom line. These sharp changes in *t* and *Q*_6_ becomes more pronounced as the path is closer to the critical pressure. Adapted from Reference [34].

Similarly to what was observed for the thermodynamic properties, we see a sharp transition, from those resembling the LDL phase to those resembling the HDL phase when the system crosses the Widom line, in the translational *t* (Figure 4(a)) and orientational order parameters *Q*_6_ (Figure 4(b)). These sharp changes in *t* and *Q*_6_ becomes more pronounced as the path is closer to the critical pressure.

### 2.4. Dynamics Crossover and Liquid-Liquid Phase Transition

**Figure 5.**
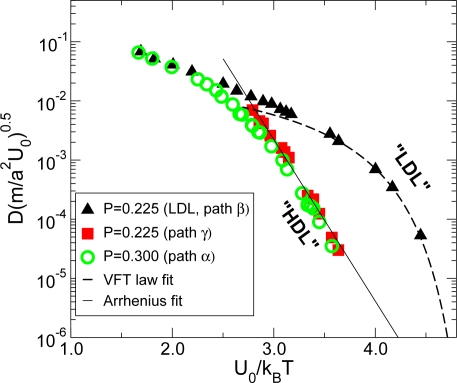
Dynamic behavior for Jagla ramp potential. The *T*-dependence of the diffusivity *D* along constant pressure paths: (i) *P* < *P_c_* for path *β* (LDL) and path *γ* (HDL), and (ii) *P* > *P_c_* for path *α*. Along path *β*, the liquid remains in LDL phase due to metastability, and its diffusivity follows a Vogel-Fuchler-Tamann (VFT) fit, indicating a fragile liquid. Along path *γ* in the HDL phase, the liquid is strong, indicated by the temperature independent activation energy. For the path above the critical point, the system shows a crossover upon crossing the Widom line, from LDL-like at high temperature to HDL-like at low temperature side. The dashed line is a Vogel-Fulcher-Tamann fit. Adapted from Reference [34] with a correction of normalization factor of 600 in the value of *D* by Corradini *et. al* [90].

In the region of the P-T phase diagram between the LDL and HDL spinodals, the system can exist in both the LDL and HDL phases, one stable and one metastable. Along path *β* (Figure 2), the LDL phase remains metastable before crossing the LDL spinodal line. The dynamic behavior of the less ordered LDL phase follows the non-Arrhenius Vogel-Fulcher-Tamann (VFT) law (Figure 5, Triangle up), which is the characteristic of fragile glass formers. On the other hand, along paths *γ* which belong to the HDL phase, *D* follows Arrhenius behavior (Figure 5, Square), which is the characteristic of the strong glass formers.

For *P* > *P_c_* along path *α*, there is a crossover in the behavior of *D* (Figure 5, Circle). The behavior is similar to what was observed in experimental studies of the strong liquid BeF_2_ [89], confined water [24] and in simulations of SiO_2_ [31]. In both cases, the Arrhenius slope extrapolates to an intercept at 1/*T* = 0, which is six orders of magnitude above the intercept of the high temperature Arrhenius part of the plot (which is common to all phases). Thus, the behavior of the HDL-like liquid on the low-temperature side of the Widom line can be classified as that of a strong liquid. The behavior on the high-temperature side of the Widom line, in the LDL-like phase, however, is very different, resembling that of the fragile liquid, as is clear from Figure 5. Thus, the present spherically-symmetric Jagla ramp potential exhibits a dynamic crossover from fragile liquid (LDL-like) at high-temperature to strong liquid (HDL-like) at low-temperature, suggesting the analogous fragile-to-strong transition as in water. We note that the strong liquid for Jagla potential is now the HDL phase, while the strong liquid for water is the LDL phase, due to the fact that the coexistence line for Jagla potential is positively sloped.

The Jagla ramp potential has an accessible LLCP and also displays water-type thermodynamic- and dynamic- crossover which occurs as the system crosses the Widom line while cooled along constant pressure paths *P* > *P_c_*. These results, similar to simulations of silicon [31], show that the dynamics is Arrhenius in the more ordered phase (HDL for Jagla ramp model) and fragile for the less ordered phase (LDL for Jagla ramp model). The dynamic crossover for *P* > *P_c_* is consistent with (i) the experimental observation in confined geometries (small pores) of a fragility transition [62], and (ii) experimental observation of a peak in the specific heat upon cooling water at atmospheric pressure in nanopores [60]. The existence of a single-component, monoatomic, system with two distinct glassforming liquid phases, provides a rare opportunity for study of fundamental aspects of glass formation.

## 3. Liquid—Glass Transformations

Despite being monatomic, and also spherically symmetric in its interaction potential, the Jagla system proves vitrification during cooling at rates that are very moderate by simulation standards. The observation of a LLPT in the Jagla model [30,34,35,37–39] suggests that two different glasses should exist at low temperature. The high density liquid (HDL) is expected to transform into a high density amorphous (HDA) solid upon isobaric cooling at *P* > *P_c_* [path *α* in Figure 2]. Similarly, the low density liquid (LDL) should transform into a low density amorphous (LDA) solid upon cooling at *P* < *P_c_* [path *β* in Figure 2].

### 3.1. Low Density Amorphous and High Density Amorphous

It is well known that the vitrification of monatomic liquid cannot be assumed even in simulations [17,91] since crystallization will usually occur during the cooling process. We find that HDA can indeed be formed if the liquid is cooled at a “slow” rate at *P* > *P_c_* (path *α*) [43]. However, cooling the liquid at *P* < *P_c_* (path *β*) at the same rate results in crystallization. A faster (“intermediate”) rate [43] is required in order to obtain LDA. We note that upon cooling along path *β*, the liquid with LDL-like local geometry crosses the LLPT coexistence line (Figure 2). However, the LDL-to-HDL spinodal is never crossed so the system remains in the LDL phase due to metastability. Therefore, further cooling leads to vitrification of solid LDA without HDL formation.

Upon cooling the liquid at *P* > *P_c_* (path *α*), although the system is in the one-phase region, a smooth crossover (*not* a transition) occurs from more LDL-like local geometry at temperatures well above the Widom line *T* > *T*_W_ to more HDL-like local geometry well below the Widom line [30] (Figure 2). The structural heterogeneity that characterize the Jagla liquid are such that for *T* > *T*_W_ the system can be thought of as a sea of molecules with locally LDL-like geometry, in which isolated molecules (and small clusters of molecules) with locally HDL-like geometry appear. As *T* decreases, the clusters of molecules with locally HDL-like geometry increase in number and size until there is a crossover at *T*_W_. For *T* > *T*_W_, the system can be thought of as a sea of molecules with locally HDL-like geometry, in which only isolated molecules (and small clusters of molecules) with locally LDL-like geometry occur. Thus one observes vitrification of the liquid with HDL-like local geometry to HDA.

**Figure 6.**
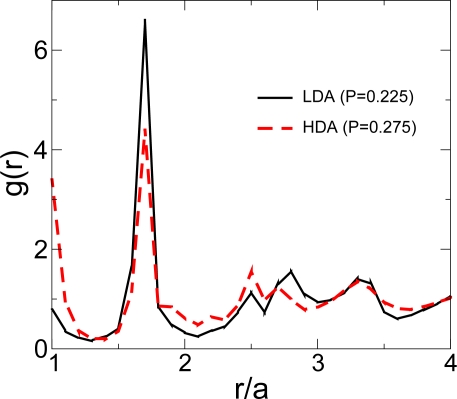
Illustration of the structural difference of the low density amorphous (LDA) solid and the high density amorphous (HDA) solid by the radial distribution function *g*(*r*). For LDA, there are more particles sitting near the soft-core distance; while for HDA, particles shift from the soft core distance (*r/a* = 1.72) to the hard core distance (*r/a* = 1.0), so the peak at *r/a* = 1.72 decreases while the peak at *r/a* = 1.0 increases. Adapted from Reference [43].

Figure 6 compares the radial distribution functions (RDF) of LDA and HDA obtained along *P* < *P_c_* and *P* > *P_c_*, respectively. Both RDFs are clearly different indicating that LDA and HDA are indeed distinct glass phases. For LDA, the majority of the particles are located around the soft-core distance, in the vicinity of the minimum of the pair potential (corresponding to the peak of the RDF at the soft-core distance *r/a* = 1.72 in Figure 6). For HDA, neighbors are observed at both the hard-core distance (*r/a* = 1) and the soft-core distance. The present results suggest that the presence of two scales in a pair interaction potential can be sufficient for a system to be a good glass former.

### 3.2. Pressure Dependence of the Glass Transition

**Figure 7.**
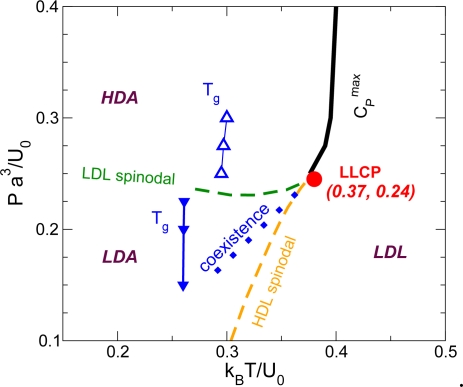
Phase diagram in liquids and glass states. The HDL and LDL have different glass transition temperatures, clearly indicating two types of glasses in system with liquid-liquid phase transition.

In this system, the glass transition temperature, *T_g_*, determined by differential scanning calorimetry, is weakly *P*-dependent, similar to the observation in metallic glasses. The line of *T_g_* is continuous within either the LDL or HDL phases [43]. However, it shows a discontinuity as the LDL spinodal line is crossed (Figure 7). This interesting result can be used in experiments to test whether a liquid presents polyamorphism. For example, in some substances such as water, crystallization occurs just above *T_g_*. In this case, isothermal compression of LDL into HDL cannot be performed at *T* ≈ *T_g_* since crystallization can occur. Thus, the presence of polyamorphism cannot be tested close to *T_g_* by compression of LDL. In this cases, measuring *T_g_* at different pressures and identifying a discontinuity would indicate that polyamorphism in the glass state extends above *T_g_* to the liquid phase [92].

### 3.3. Density Minimum and Glass Transition

**Figure 8.**
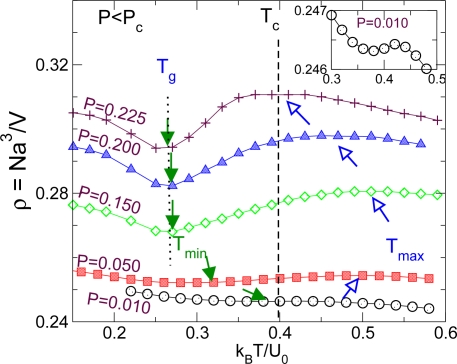
Demonstration of density minimum is affected by the glass transition along path *β* below *P_c_*. For low pressures the temperature of minimum density is located in ergodic region, approaching to the temperature of maximum density (see inset). For relative high pressures below *P_c_*, the density minima are preempted by glass transition, indicated by the same location of the density minimum along different *β* paths. Adapted from Reference [43].

Another important question—especially relevant for liquids with density anomalies such as water, BeF_2_, Si, and SiO_2_—is how the anomalous thermal expansion behavior upon cooling below the temperature of maximum density *T*_max_ in the supercooled liquid changes to “normal” behavior in the glass state. The display of a temperature of maximum density is a striking feature of the Jagla model, as described earlier [30,34,35]. What is more remarkable is the existence of the even rarer density minimum [43], which makes studies of the Jagla model useful for the understanding of the general relations between the density anomaly and LLPT. This feature, has been seen not only in experiment of confined water [26,27] and in simulation of bulk water [93,94] but also in supercooled Te, stable As_2_Te_3_ [95] and some Ge-Te alloys [96], and at the upper limit of experiments for BeF_2_ [89] and in silica [97]. In Reference[43], Xu *et. al.* showed how these features are unique to the low density polymorph and vary in a complex way with pressure. As shown in Figure 8, the density maximum is an equilibrium property, but the density minimum in the equilibrated liquid is only seen at the lowest pressures, in the temperature range between the *T_g_* and the *T_max_*. At relatively higher pressures below the critical pressure *P_c_*, the density minimum is preempted by the GT for the cooling rates applied. For slower cooling rates [43] the minimum would presumably continue to be seen as an equilibrium phenomenon.

## 4. Conclusions

In this review, we have discussed the phase transformations in the Jagla model, which was parametrized in order to show polyamorphism at high temperature in the equilibrium liquid phase. The presence of a LLCP results in a sharp increase in thermal (e.g., *C_P_*) and structural response functions upon cooling as the Widom line temperature *T*_W_ is approached. Such a sharp increase in thermal response functions is anomalous (*i.e.*, in normal liquids, *C_P_* decreases upon cooling) and is observed in few substances such as water [98]. It is therefore indeed reasonable to assert that the anomalous behavior of bulk water seen at normal and moderate pressures can be associated with the presence of a nearby LLCP and also helpful to look for comparable behavior in other systems.

Further, the Jagla model proves to have not only one but two very different liquids, which vitrify to different glasses upon cooling with rates common in computer simulations. These glasses are different amorphous forms both from the structural (e.g., their RDFs are distinct) and thermodynamics point of view (e.g., their *T_g_* values are different). In particular, we observed that *T_g_* is practically constant for each glass but it is larger for HDA than for LDA. *T_g_* changes discontinuously (by ≈ 17%) as we go from LDA to HDA across the transition line. The study of the relation between LDA and HDA and the possible transformations between each other are relevant to understand polyamorphism in the glassy state [1,2].

Lastly, we note that the Jagla model was originally proposed to model water-like anomalous behaviors [37–39,83]. However, with a different parametrization than that one used here, it might be a good candidate to model cerium. Cerium crystallizes into HCP at low pressure, as the Jagla model does, and Ce-Al alloys show polyamorphism in the glass state [19]. It will be interesting to see if the Jagla model can be parametrized to yield other properties particular to cerium, such as its isosymmetric crystal-crystal (FCC-FCC) transition [99]. It is then understandable that the glass formation in cerium-based alloys is only obtained with multiple component doping, or very rapid quenching, as reported in the recent literature [100].
